# A Scientific Achievement of Practical Value

**Published:** 1887-11

**Authors:** 


### A SCIENTIFIC ACHIEVEMENT OF PRACTICAL VALUE.

Pictet & Cailletet, a firm of French chemists, started out with the assumption that all gases could be reduced to liquid or solid form, under pressure at a low temperature—and that it was only a question of how much pressure, and how low a temperature, was required to liquify a given gas. They accordingly constructed the necessary machinery, and, in 1877, succeeded in reducing to liquid form all the known gases, carbonic acid gas being among the last to yield to pressure.

It is found that under a pressure of 36 atmospheres, and at a certain temperature, carbonic acid gas (carbon di-oxide) readily liquifies; and it is now bottled in wrought iron cylinders, which can be handled or shipped in any climate, with absolute safety, and furnished to soda water manufacturers, and others who bottle carbonated drinks.

This discovery will produce a complete revolution in certain industries, notably, the soda water manufacture. It does away with, completely, first, the complicated, costly and dangerous machinery heretofore used for liberating carbonic acid gas from the carbonates by means of an acid,—does away with both the carbonate and the acid, and reduces the matter to a very simple and safe procedure. All that is necessary to do, to charge the water or other liquid to be carbonated, is to connect the cylinder containing the liquid carbonic acid, with the fountain, and open a valve, when the gas will stream through the rubber connecting tube,—there being a gauge attached also, which will indicate the pressure, as in the old style. When the requisite pressure is obtained, disconnect the cylinder and agitate the fountain, when the process of charging will be complete. The quantity that can thus be stcred in one cylinder is immense, and will serve to charge a great many fountains.

The same principle has long since been put into practice in making ice, by the Carre process. The ammonia, instead of being obtained from aquæ ammonia by heat, as at first done when the Carre process was introduced, is now furnished in the form of ammonia gas, liquified under pressure; and quite recently a mixture of compressed (liquified) sulphurous acid gas and carbonic acid gas, has been successfully tried in the manufacture of ice, as its re-evaporation produces the most intense oold.

The compressed carbonic acid gas is certainly destined to come into general use, being so very cheap, and to render worthless the many forms of "generators" devised for ærating fluids, and which cost from $175 to $1000 each. This is truly a scientific achievement of practical value.

There is a well known carbonic acid gas well in Germany, where formerly the gas was allowed to escape; but it has now been utilized. Machinery having been erected, the gas is compressed as it comes out, and is shipped to all parts of the world, securely bottled in wrought iron cylinders of portable size. Thus, the chemist has scored another invention of practical value.

